# Sand Quality on Portuguese Blue Flagged Beaches: Fungal and Faecal Contamination Across Two Bathing Seasons

**DOI:** 10.3390/microorganisms14051043

**Published:** 2026-05-05

**Authors:** Ana Margarida Silva, Konstantina Sarioglou, Susana Silva, Carla Viegas, Edna Ribeiro, Maria Teresa Rebelo, João Brandão

**Affiliations:** 1 National Institute of Health Dr. Ricardo Jorge, 1649-016 Lisboa, Portugal; anamargaridasilva134@gmail.com (A.M.S.); konstantina.sarioglou@insa.min-saude.pt (K.S.); susana.pereira@insa.min-saude.pt (S.S.); 2 Health & Technology Research Center (H&TRC), Escola Superior de Saúde (ESSL), Instituto Politécnico de Lisboa, 1990-096 Lisbon, Portugal; carla.viegas@essl.ipl.pt (C.V.); edna.ribeiro@essl.ipl.pt (E.R.); 3 Public Health Research Center, Comprehensive Health Research Centre (CHRC), Research Unit of the Associated Laboratory (REAL), Centro Clínico Académico de Lisboa (CCAL), National School of Public Health NOVA University Lisbon, 1600-560 Lisbon, Portugal; 4 Centre for Ecology, Evolution and Environmental Changes (CE3C), Global Change and Sustainability Institute (CHANGE), Faculdade de Ciências da Universidade de Lisboa, Campo Grande 016, 1749-016 Lisboa, Portugal; mtrebelo@ciencias.ulisboa.pt

**Keywords:** beach sand, enterococci, *E. coli*, fungi, monitoring

## Abstract

There is growing concern about the quality of sand on beaches, as users tend to spend most of their time on the sand rather than in the water. Numerous pathogenic agents have reportedly been isolated from sand, including bacteria, nematodes and opportunistic fungi. The ability of sand to retain pollutants and facilitate the transmission of pathogens raises public health concerns. We analysed sand-monitoring data from the 2024 and 2025 bathing seasons on Blue Flag beaches to find trends and patterns in total fungal counts, enterococci, and *E. coli*. The values recorded for microorganisms showed considerable variability, which may reflect the possible combined influence of multiple climatic, environmental, and anthropogenic factors contributing to their presence in beach sand. Our findings suggest that the total fungal count on coastal beaches may be influenced by periods of rainfall, which increases the fungal load in the sand. Values recorded from inland beaches vary considerably between beaches which may reflect the influence of local environmental characteristics, particularly vegetation and beach morphology, although the smaller number of inland samples also makes it difficult to define clear patterns and consistent reference values for this parameter. Bacterial indicators may be particularly influenced by occasional anthropogenic disturbances and contamination events. This study adds significantly to the understanding of the microbiological quality of beach sand, encouraging the integration of sand monitoring into environmental surveillance and management programmes.

## 1. Introduction

There is increasing concern about the quality of sand on recreational beaches, as people spend most of their time on the sand rather than in the water [1]. It is on the beach sand that visitors lay their towels, children play, and various recreational activities take place. Wind can also facilitate the deposition of sand grains in natural orifices and mucous membranes, on the skin, ears, mouth, and hair, as well as their inhalation or ingestion, making beach sand an object of interest regarding its quality and other factors that may, in some way, impact the health and well-being of its users [2,3]. 

Beach sand, which is typically composed of various unconsolidated sediments and water, may incorporate a wide range of pollutants and microorganisms, such as bacteria, viruses, and fungi, some of which exhibit pathogenic activity [4,5]. Bacteria in beach sands have been widely studied due to their potential public health risks. Species such as *Vibrio vulnificus* and *Pseudomonas aeruginosa* may occur naturally in these environments, whereas others including *Campylobacter* spp., *Salmonella* spp., and *Staphylococcus aureus* are commonly associated with faecal contamination or human skin and mucous membranes and may cause infections ranging from gastrointestinal illness to skin and ear infections [6,7,8,9]. Contact with contaminated sand has also been associated with an increased risk of gastrointestinal illness among beach users, particularly children who dig or are buried in sand [10,11]. Fungi present in beach sands are predominantly saprophytic organisms involved in decomposition and nutrient cycling, although their distribution remains poorly understood [12,13]. While only a small proportion of fungal species are capable of infecting humans, largely due to physiological constraints such as the ability to grow at body temperature (≈37 °C), some clinically relevant fungi have been isolated from sand, including dimorphic pathogens (Histoplasma, Coccidioides, Blastomyces), opportunistic species (*Candida albicans*, *Aspergillus fumigatus*, *Cryptococcus* spp.), and dermatophytes (*Trichophyton*, *Microsporum*), which may cause respiratory, systemic, or cutaneous infections [2,14,15,16,17,18,19,20,21,22]. Despite their global health impact, fungal diseases remain relatively overlooked, even though they may lead to infections, allergic reactions, and irritation [23,24,25].

To monitor the possible presence of pathogenic microorganisms in recreational waters, *E. coli* and enterococci are commonly analysed. These are not necessarily pathogenic, but they provide a practical indication of faecal contamination [26]. Although bathing-water quality is a well-established criterion, the potential relationship between sand quality and water quality has only gained recognition more recently [26]. Through runoff, tidal action, waves, or even human transport, seawater and sand in the intertidal zone form a dynamic interface characterised by intense interaction and potential accumulation of microorganisms [27,28,29,30,31]. Despite the constant interaction, multiple studies have shown that these microorganisms are often present in higher abundance and concentration in sand than in adjacent bathing waters due to the greater capacity of sand to retain these microorganisms and its porous structure that retains organic matter acting as a substrate [32,33,34,35,36,37,38]. In addition, several studies indicate that direct exposure to beach sand constitutes a risk factor for infectious diseases, given the confirmed presence of pathogenic microorganisms, highlighting the need to investigate and monitor the microbiological quality of beach sands [39,40,41]. 

Both the 2006 European Union Bathing Water Directive and the 2012 criteria of the United States Environmental Protection Agency (USEPA) recognise sand as a potential source of faecal contamination for adjacent waters; however, the importance of sand contamination alone is not yet recognised [42]. Argentina was the first country to formally include sand inspection in its national bathing-water regulation in 2017 including *E. coli* and enterococci as faecal contamination indicators, highlighting the importance of sand quality itself [43]. The World Health Organization recognised the potential public health risks associated with microorganisms in beach sands in the 2021 update of the Guidelines for Safe Recreational Water Environments. These guidelines highlight the presence of pathogenic bacteria and fungi previously detected in beach sands, including *Staphylococcus* spp., *Pseudomonas aeruginosa*, *Campylobacter jejuni*, *Trichophyton mentagrophytes* and *Candida albicans*, and encourages sand monitoring through recommended sampling approaches, analytical methods and management actions. In addition, guideline values were proposed for enterococci [32]. Overall, awareness of the importance and efforts to monitor recreational beach sands have gradually evolved over time, in parallel with advances in scientific knowledge and public health policy [44,45,46,47].

The ABAAE (Blue Flag Association for Environmental Education) is an international environmental certification initiative aimed at promoting environmental quality, safety, sustainable management, and environmental education in beaches, marinas, and boating tourism. The strong summer seasonality of Portuguese coastal tourism, combined with intense tourist demand and urban growth, increases the risk of pollution events and highlights the need to ensure environmental preservation and user safety [48,49]. In 2024, the Blue Flag added sand monitoring of at least one beach per municipality as a mandatory criterion for award attribution [50]. This criterion includes sampling methods designed to represent the entire dry beach zone [51] and establishes limits of 420 CFU/g for total fungal counts in coastal beaches and 1130 CFU/g in inland and transitional beaches. These values are based on the 80th quartile of the guidance value of 89 CFU/g [2], with only 20% of collected samples permitted to exceed this guidance threshold. For enterococci, the maximum value of 60 CFU/g is indicated based on an exposure risk assessment calculation in which 60 CFU/g is considered equivalent to 200 CFU per 100 mL of water, corresponding to a probability of illness from exposure of at most 5% of beach users [32]. This value, as stated by the WHO [26], reflects the same health effects as water-based limits. However, it is considered provisional because it derives from a Quantitative Microbial Risk Assessment (QMRA) calculation that does not account for a beach’s native flora. The *E. coli* limit of 25 CFU/g is a reference value [51]. Mandatory sand monitoring for award attribution places Portugal among the pioneers in recognising the importance of sand for public health and represents an important step toward a better understanding of this issue. This approach also reflects the need for integrated beach management considering emerging challenges such as climate change and microbiological risks, that are interlinked [52]. In addition, monitoring and subsequent assessment serve as an early warning system, contributing to awareness of public health risks, reinforcing commitment to environmental quality and population safety [3].

The primary aim of this study was to assess the microbiological quality of Portugal’s beach sands during the 2024 and 2025 bathing seasons at beaches applying for the Blue Flag award, in order to identify potential trends and contamination patterns associated with the three analysed parameters: total fungal counts, enterococci and *Escherichia coli*. To achieve these objectives, this study relied primarily on classical microbiological techniques and descriptive statistical analyses. In addition, it aimed to discuss known potential contamination sources, as well as environmental and anthropogenic factors that can influence the levels of microorganisms detected on the sand. A further objective was to characterise the presence of fungi in these recreational environments, highlight their potential implications for public health and to reinforce the importance of integrating fungi alongside faecal indicators in beach sand monitoring programmes.

## 2. Materials and Methods

### 2.1. Study Area and Sample Collection

This study analysed the results of the microbiological quality assessment of Portuguese beaches awarded the Blue Flag. Sand samples from 122 beaches were analysed in 2024 bathing season and 149 in 2025 (May to September), with some additional samples collected in April and October.

Sampling procedures and laboratory methods were established by ABAAE and are set out in the Blue Flag criteria, which describes how the sampling and the three analysed components should be assessed. Municipalities and their respective laboratories were responsible for ensuring that all analyses were conducted in accordance with these criteria. All data were generated by laboratories complying with the technical requirements of ISO 17025 [53]; however, some degree of inter-laboratory or inter-method variability cannot be excluded.

Sampling was conducted by the respective municipalities. Sand was collected from three points within the dry beach zone at each site, at a depth of 10 cm, using gloves and sterile containers [51]. Samples were collected once per month, simultaneously with bathing-water sampling, and subsequently transported under refrigerated conditions to the designated laboratory for analysis [54], as stated by ABAAE.

### 2.2. Microbiological Analyses

For enumeration of enterococci and *E. coli*, the method defined in the Blue Flag criteria [54], allows two alternative approaches: (1) The method described in [54] consists of weighing 10 g of sand and adding 100 mL of sterile distilled water, using the Enterolert Quanti-Tray system (IDEXX™, Westbrook, MN, USA). Alternatively, (2) the method described in [51] uses 50 g of sand mixed with 500 mL of sterile distilled water and agitated at 100 rpm for 30 min. After agitation, 10 mL aliquotes are analysed and the volume completed to 100 mL with sterile distilled water. As an alternative, the membrane filtration method (ISO 7899-2) [55] may also be applied [56]. For *E. coli*, the same extraction procedures are followed using Colilert.

Given the use of the Enterolert/Quanti-Tray method, results were expressed as Most Probable Number (MPN) per gram of sand, which represents a statistical estimate of viable microorganisms based on the number of positive wells observed after incubation. MPN provides an indirect estimate of viable microbial concentration (unlike colony-forming units used for fungi (CFU), which are based on direct colony counts on solid media). Although these approaches are all accepted under the ABAAE framework and results are reported per gram of sand, they are not analytically identical. Differences in extraction and detection procedures may influence bacterial recovery and quantification, and complete methodological homogeneity therefore cannot be assumed.

For the small subset of fungal analyses for which the authors were directly responsible, the following procedure was applied. This procedure followed was defined in the ABAAE criteria in [51,54].

For total fungal enumeration, 40 g (gross weight) of homogenised sand were weighed into sterile Schott bottles to which 40 mL of sterile distilled water were added. The mixture was agitated orbitally for 30 min at 100 rpm (Orbital agitation was used throughout the procedure to avoid hyphal fragmentation, as each fragment may originate an independent colony, which could artificially increase the fungal counts.

Under laminar flow conditions, 200 µL from each sample were spread, in triplicate, onto Petri dishes. The Petri dishes were incubated for 5–7 days at 27.5 ± 2.5 °C. After this period of incubation, total fungal colonies from the three plates were counted macroscopically (Figures S3 and S4) The mean of the three replicates was calculated and expressed as colony-forming units per gram of sand (CFU/g; assuming 1 mL = 1 g) [2,51,54]. Additionally possible replicate rejection followed the Lightfoot table method (one replicate was excluded if falling outside the acceptable range) [57].

The medium used for fungal growth contained malt extract agar supplemented with chloramphenicol (Oxoid, Milan, Italy), which supports growth of a wide range of fungi while inhibiting bacterial growth due to the added antibiotic. This medium is also nutritionally suitable for diverse species and may slow growth of fast-growing fungi, reducing colony size and facilitating colony counting [58].

Given the scope of the study most of the analytical data were generated by external laboratories and personnel. Although the authors were responsible for the processing of part of the samples, all final results were submitted to the ABAAE platform and analysed as end-point values only; however, all results and the corresponding analytical methods are traceable to their respective laboratories.

### 2.3. Data Analysis

Data recorded included total fungal counts, *E. coli*, and enterococci levels, sampling date, beach name, municipality, region, and beach typology (coastal or inland).

Results were registered on the Blue Flag platform, which provided the dataset for this study.

Meteorological data were also collected, including maximum temperature recorded in the seven days preceding sampling and relative humidity at the sampling location. Data were obtained from three sources [59,60,61] to ensure completeness. Relative humidity was calculated either from dew point (RH = 100 × e (Td)/e (T), where T is air temperature and Td is dew point) [61] or as the daily mean relative humidity provided by the source [60]. Monthly mean temperature and total precipitation were also recorded [59]. Data were obtained from regional meteorological stations, which may not capture municipality-level variability.

Data were initially stratified by beach type (coastal, inland and transitional) sampling month, and region (Azores, Madeira, North, Centre, Lisbon and Tagus Valley, Alentejo, Algarve). Due to limited sample numbers in April and October in 2024, these months were grouped with May and September, respectively (April/May and September/October), forming pre-season and post-season groups. Transitional beaches were grouped with inland beaches due to their limited number and consistent with Blue Flag criteria.

Statistical analyses were performed using R version 4.5.1. Descriptive analyses were conducted by beach type and region, including sample size, medians, first and third quartiles, and minimum and maximum values for the three parameters. The median values were calculated from all individual samples recorded for the corresponding group, including zero values.

Non-parametric tests were applied as appropriate: the Mann–Whitney–Wilcoxon test for comparisons between two groups and the Kruskal–Wallis test for three or more groups. Statistical significance was set at *p* < 0.05.

Graphical representations including boxplots and line graphs were generated. Upper limits were applied to some graphs to minimise distortion by extreme outliers and better clarity. Pearson correlation coefficients were calculated to assess the possible existence of a linear relationship between the analysed continuous quantitative variables such as maximum temperature in the previous seven days, relative humidity on sampling day, and the three analysed microbiological parameters of the study.

As a preliminary assessment of performance, the percentage of samples exceeding the limits given by the Blue Flag, was calculated for identification of non-compliant samples and to provide an initial overview of contamination status. In addition, an exceedance situation was considered when more than 20% of the samples exceeded the established limits and when more than one parameter exceeded the limits within the same sample, in line with the threshold suggested by ABAAE.

## 3. Results

In 2025, Portugal recorded an increased number of beaches awarded the Blue Flag, 404 compared to the 398 awarded in 2024. This places Portugal in 6th place among 51 countries worldwide within this programme of environmental excellence [62]. Although Blue Flag criterion requires sampling in the pre-bathing season in May and monthly sampling throughout the bathing season (from June to September), implementation of this requirement was still in a consolidation phase in 2024. This was reflected in the limited number of beaches that effectively carried out all the required sampling in 2024. Despite the Blue Flag programme having standardised the bathing season period (June to September), it is important to note that this timeframe may not fully coincide with the operational reality of all municipalities of the country. The results showed a predominance of samples collected in June, July, and August compared to the other months. In addition, a predominance of samples from coastal beaches was also observed due to the fewer number of inland beaches awarded the Blue Flag and the consideration that not all inland beaches present characteristics compatible with the type of analysis undertaken, as many lack defined sandy areas or present different types of sediment, such as soil or gravel [54]. These differences in sampling across bathing seasons, as well as variations in the analysed beaches, limit the possibility of making a direct comparison between 2024 and 2025 for part of the dataset. Furthermore, the fewer samples collected from inland beaches resulted in lower statistical power compared with coastal samples.

Within the scope of the new sand quality monitoring component, only 122 beaches were analysed in 2024, and a total of 565 samples were collected. Analysis of the 2024 beach data revealed differences in the number of samples collected from coastal, inland, and transitional beaches. Coastal beaches accounted for 513 samples, inland beaches for 47 samples, and transitional beaches for 5 samples, the latter corresponding exclusively to Árvore Beach in the Northern region. For the sake of simplification, these transitional beach samples will hereafter be referred to as inland beaches.

In 2025, the beaches analysed underwent some changes compared to the 2024 dataset, with some beaches entering or leaving the dataset and a total of 149 beaches carrying out the analyses proposed by the Blue Flag Programme. The transitional beach included in the 2024 dataset was not present in the 2025 data. The number of samples collected this year was 730, with 681 samples from coastal beaches and 49 from inland beaches.

### 3.1. Fungal Counts

The total fungal count data revealed a statistically significant difference between inland and coastal beaches (*p* < 0.001), in both seasons, with median values of 330 CFU/g and 10 CFU/g for 2024, respectively (Table 1). The maximum and minimum values, as well as the first and third quartiles for inland beaches, were all higher than those recorded for coastal beaches. The maximum value observed was 44,000 CFU/g at inland beaches and 8000 CFU/g at coastal beaches (Table 1), both exceeding the established limits. However, the maximum values observed in 2025 were lower, reaching 14,000 CFU/g at inland beaches and 6500 CFU/g at coastal beaches (Table 1).

In mainland Portugal, the recorded median values for 2024 were 140 CFU/g (North), 39 CFU/g (Centre), 9 CFU/g (Tagus), 5 CFU/g (Alentejo), and 5 CFU/g (Algarve). The North region stands out, presenting the highest median and highest first and third quartile values (44 CFU/g and 326 CFU/g, respectively) when compared with the other regions. Regarding non-compliance of the established limits by region in coastal beaches, the North and Tagus regions recorded the highest number of non-compliant samples (10 (37%) and 9 (33.3%), respectively). However, it is important to highlight that the North region had a substantially lower total number of samples compared to the Tagus region (47 and 152 samples, respectively) (Table S1). For 2025, the regional medians were 65.0, 30.0, 9.5, 8.0, 3.0, 7.0 and 2.0 CFU/g for the North, Centre, Tagus, Alentejo, Algarve, Azores, and Madeira regions, respectively, once again highlighting the North and Centre regions in comparison to the rest of the country (Table S2)

For inland beaches, only the North, Centre, Alentejo, and Tagus regions had samples. The North region again stood out, with a median value above the defined threshold (2200 CFU/g), with 11 of the 18 analysed samples contributing to this result (Table S1). In 2025 median values exceeding the established thresholds were observed in the North and Alentejo regions (1950 CFU/g and 2200 CFU/g, respectively) (Table S2).

In this year, all inland beaches in these two regions exceeded the defined limits at least once during the analysed period. The Kruskal–Wallis test indicated statistically significant differences between regions for this beach typology (*p* < 0.001).

**Figure 1:**
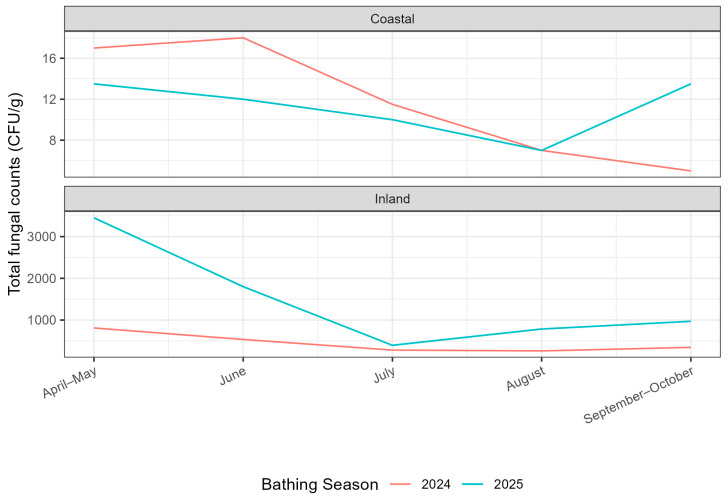
Medians of the Total fungal counts (CFU/g) in Coastal and Inland Beaches over the course of the 2024 and 2025 bathing seasons.

An analysis of the variation in total fungal counts across the sampling months of 2024, April and May (grouped), June, July, August, and September and October (grouped), indicates that, for coastal beaches, the median values remained relatively stable throughout 2024 (17, 19, 11, 6, and 5 CFU/g, respectively). However, when comparing the different monthly groups, a statistically significant difference was identified in the distribution of counts among the months (*p*-value = 0.046). A gradual decrease in median values, as well as in the first and third quartiles, can be observed over the course of the sampling months. April and May (pre-bathing season) recorded the highest values when compared to the months within the official bathing season (Figure 1).

This was also observed in 2025. The median value during the pre-bathing season (May) was 13.5 CFU/g, followed by a gradual decline throughout the bathing season, with August contributing to the lowest recorded median value (7 CFU/g), and an increase again in the post-bathing season. However, these variations were not statistically significant (*p* = 0.153) (Figure 1).

Regarding inland beaches, no statistically significant differences were observed in 2024 between the sampling months (*p* = 0.517). The recorded median values were 810 CFU/g, 535 CFU/g, 280 CFU/g, 260 CFU/g, and 345 CFU/g, respectively, showing a decrease until July and August, followed by a slight increase towards the end of the bathing season. For 2025, a similar trend was observed, with no differences found between months (*p* = 0.439). The increase in median values occurred in August, with the lowest value recorded in July (395 CFU/g) (Figure 1).

Furthermore, median values for inland beaches exceeded the established threshold limits in May (3450 CFU/g) and in June (1800 CFU/g), which was not observed in the previous year.

The highest values observed in both 2024 and 2025 were predominantly associated with inland beaches. In 2024, the three highest values were observed at Praia Arquiteto Albino Mendo (North region), reaching 44,000 CFU/g, 28,000 CFU/g, and 13,000 CFU/g, exceeding the defined thresholds during three months of the bathing season, while the fourth highest value (12,000 CFU/g) was recorded at Fraga da Pegada, also an inland beach in the North region. In 2025, the two highest values were recorded at Quinta do Barco (Centre region), followed by Amieira, an inland beach in the Alentejo region, whereas the fourth highest value was observed at the coastal beach Homem do Leme in May (6500 CFU/g).

Regarding the total fungal count parameter, a higher percentage (28.8%) of inland beaches recorded samples exceeding the established threshold values in 2024, with 15 samples above the limits. In contrast, this situation occurred in only 5.3% in coastal beaches (27 samples) (Table S3).

In 2025, however, 49% of samples from inland beaches exceeded the defined limits (24 samples compared to 25 samples within acceptable levels), revealing a marked increase relative to the previous year. Among coastal beaches, non-compliances occurred in only 3.4% of cases (23 samples) (Table S4).

### 3.2. Faecal Contamination Indicators

#### 3.2.1. 2024

Enterococci values varied significantly between coastal and inland beaches (*p* < 0.001), while no significant difference was observed for *E. coli* (*p* = 0.404). The highest number of enterococci non-compliant samples occurred in the North and Tagus regions (9 and 6 out of 23, respectively). Inland beaches in the Centre region presented a high third quartile (47 CFU/g), although still below the established limits.

**Figure 2:**
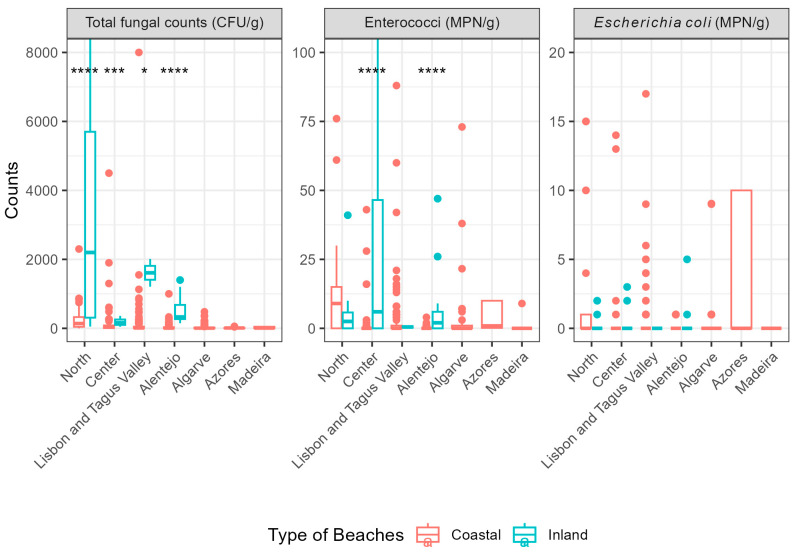
Boxplots of the 3 parameters by region in Portugal in the 2024 bathing season (truncated y-axis). Symbols indicate levels of statistical significance according to the following thresholds: *, *p* ≤ 0.05; ***, *p* ≤ 0.001; ****, *p* ≤ 0.0001.

Across all regions, beach types and sampling months, *E. coli* values remained low, with medians around 0 CFU/g. Enterococci values at inland beaches were highest in August. Overall, the observed values indicate generally low levels of faecal contamination, aside from occasional contamination events (Figure 2).

In 2024, the proportion of non-compliant samples was higher for enterococci than for *E. coli.* A total of 17 samples exceeded the enterococci limits at coastal beaches (3.3%) and 6 at inland beaches (11.5%), whereas *E. coli* non-compliant samples occurred in 8 coastal samples (1.6%) and in 1 inland sample (1.9%), all belonging to different beaches (Table S3).

#### 3.2.2. 2025

**Figure 3:**
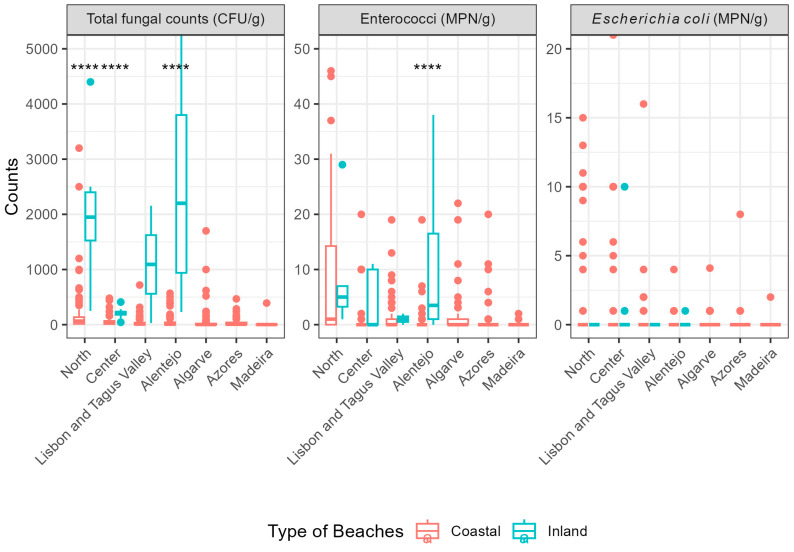
Boxplots of the 3 parameters by region in Portugal in the 2025 bathing season (truncated y-axis). Symbols indicate levels of statistical significance according to the following thresholds: ****, *p* ≤ 0.0001.

Median values were generally low. However, inland beaches in the Centre region showed a markedly high third quartile (687 CFU/g), influenced by high values recorded consistently at Quinta do Barco beach. Nevertheless, no significant difference was observed between coastal and inland beaches in this region (*p* = 0.088) (Figure 3).

Overall, non-compliances were proportionally higher at inland beaches than at coastal beaches for both parameters (enterococci: 8.2% vs. 1.8%; *E. coli*: 4.1% vs. 0.9%). Only eight samples exceeded the *E. coli* limits, with values remaining close to zero across regions and months, although the Lisbon and Tagus Valley region accounted for half of these cases (Table S4).

Median enterococci values oscillated at inland beaches across the sampling period. The values decreased until July and increased afterwards, while coastal beaches showed relatively stable values.

In 2025, non-compliant samples were again more frequent for enterococci. Sixteen samples exceeded the enterococci established limits, 12 samples from coastal beaches and 4 from inland beaches, mostly in the North and Centre regions (Table S4).

### 3.3. Exceedances

During the 2024 bathing season, 14 of the 122 analysed beaches exceeded the established limits for more than one parameter or exceeded the same parameter on two or more occasions, corresponding to 11.5% of the total beaches. Of these, eight were located in the North region (Aquário, Fraga da Pegada, Foz–Porto, Gondarém, Homem do Leme, Ribeira—Macedo de Cavaleiros, Quebrada, and Praia Arquiteto Albino Mendo), three in the Centre region (Cascalheira–Secarias, Mamoas, and São Jacinto), and the remaining three in the Lisbon and Tagus Valley region (Carcavelos, Lourinhã, and Moitas).

In 2025, beaches exceeding the established limits for more than one parameter or surpassing the same parameter on two or more occasions represented 10.1% corresponding to 15 of the 149 analysed beaches. The beaches Homem do Leme, Foz–Porto, Fraga da Pegada, Ribeira—Macedo de Cavaleiros, and Arquiteto Albino Mendo again appeared in this group. These were joined by Quinta do Barco, Praia Fluvial do Alqueva, Praia Fluvial de Reguengos de Monsaraz, Amieira, Praia Fluvial de Mourão (inland beaches), Apúlia, Sereia, Miramar, Figueirinha and Pedras do Corgo (coastal beaches). The north region was once again the region with the highest number of exceedances.

The correlation analysis conducted between sampling month, maximum temperature over the previous seven days, air humidity on the sampling day, and the three analysed parameters revealed some weak correlations. Regarding the three study parameters, a positive correlation (ρ = 0.29) was identified between enterococci and *E. coli*, as well as between total fungal count and humidity on the sampling day (ρ = 0.14) (Figure S1).

Correlation analyses performed with the 2025 data demonstrated a weak positive correlation was observed between total fungal counts and enterococci (ρ = 0.23) and *E. coli* (ρ = 0.07), and between enterococci and air humidity (ρ = 0.10) (Figure S2).

When comparing the Total Precipitation and Mean Temperature data registered in Portugal in 2024 and 2025, it is important to highlight the marked difference observed in June, which recorded substantially higher precipitation in 2024 (42.2 mm) than in 2025 (4.9 mm), whereas the remaining months of the bathing season were relatively dry in both years. Precipitation levels were also higher during the pre- and post-bathing seasons when compared with the core bathing months (Table 2).

Overall, total fungal counts were higher in inland beaches and in pre-bathing season in coastal beaches. Faecal indicators revealed low levels, aside from occasional contamination events.

## 4. Discussion

### 4.1. Variability and Potential Drivers of Fungal Counts

Fungi are present in all environments, including beach sands in both coastal and freshwater environments. Consequently, they are naturally present in all analyses and samples collected from beach sand [63,64,65,66]. It is therefore important to emphasise that it is unlikely for a beach to present a total fungal count of zero, as observed in some of the beaches analysed in this study. This could possibly indicate inconsistencies in the sampling procedure or that the beaches to which these samples belong to have undergone sand sanitation, a practice that is strongly discouraged according to the guidelines of the World Health Organization [26].

Although there is still limited knowledge about the composition of fungal communities in these environments, it is known that these communities are expected to vary depending on the characteristics of the surrounding environment [12,29,63,64,67,68]. Inland beaches, located in freshwater environments such as lakes and rivers, exhibit physical and ecological characteristics that favour the accumulation of organic matter in sediments. Proximity to vegetation from forests or agricultural fields contributes to a more substantial direct deposition of leaves, branches, and plant debris onto the sand compared with coastal beaches. At the same time, the sediment composition, which may retain more moisture and the lower hydrodynamic energy characterised by weaker waves and currents than in marine settings, facilitates the deposition and retention of organic matter [69,70,71,72]. As a result, inland and river beaches function as reservoirs of particulate organic matter, increasing the likelihood of higher microorganism concentrations together with lower salinity and a higher likelihood of biofilm development in deeper sediment layers [73,74,75,76]. This may explain the higher fungal counts recorded in beaches of this typology in the present study and the clear distribution of higher values (including medians and Q3 values above the proposed limits) associated with inland beaches (*p* < 0.001) (Table 1), reinforcing results previously reported [2,73].

Additionally poor management of the artificial sand may be one of the causes of the contamination problems recorded at several inland beaches presenting high fungal counts [77,78,79,80,81]. This may be related to the proximity of sand areas to dense vegetation (North inland beaches being an example of this), the presence of very fine sand layers that allow contact with deeper layers containing higher organic matter, or simply the progressive erosion of sediments throughout the bathing season [70,77,78,79,80,81,82]. A meeting with the beach managers of an inland beach in 2025 clarified that sand nourishment took place in June, supporting this hypothesis that artificial sand erosion may be contributing to the elevated fungal counts or faecal indicators in that beach or others.

In addition, fungal communities adapt to variations in temperature and precipitation [2,63]. Climatic trends observed in 2024 and 2025 suggest that precipitation may be a determining factor influencing total fungal counts, particularly at coastal beaches (Figure 1). In 2024, higher median fungal counts at coastal beaches were observed in April, May, and June, which also corresponded to the months with the highest precipitation (43.5 mm, 33.5 mm, and 42.2 mm, respectively) (Table 2). Similarly, in 2025 the highest median values for both coastal and inland beaches were recorded in May, coinciding with the highest precipitation of the analysed period (42.3 mm), whereas June 2025 showed much lower precipitation (4.9 mm vs. 42.2 mm in 2024) and fungal median values comparable to those observed in July and August. Moisture retained in sand grains and sediments is essential for microorganism survival and proliferation in these environments, allowing them to survive in the sand for several months after their introduction. Fungi benefit especially from moisture retention within sand pores, which confers persistence superior to that of many bacteria [27,64,83,84,85,86]. Additionally, runoff during rainfall events has been identified as one of the most relevant sources of diffuse pollution, contributing high microbiological loads from surrounding environments [28,30].

For inland beaches this pattern is partially observed with lower median values in the driest months and an increase when higher precipitation levels were recorded. However, the difference in the distribution of these values is not significant (*p* = 0.517 for 2024 and *p* = 0.439 for 2025), suggesting a possible greater influence of the local environmental characteristics in the registered values, as previously discussed. Nevertheless, it should be noted that comparisons between these two typologies and the registered patterns are to be made with caution, as the number of inland samples is relatively limited.

Many microorganisms present in these environments may also enter a dormant state making detection by conventional laboratory methods difficult. However, with increased moisture following rainfall events, these microorganisms may resume metabolic activity and multiply, possibly resulting in the higher counts in September and October following the dry season [12,27,64].

Microorganisms are also strongly influenced by temperature and exposure to ultraviolet (UV) radiation. July and August showed the lowest median values for total fungal counts and, considering that exposure of microorganisms to UV radiation causes protein denaturation and damage to genetic material, the high temperatures recorded during these months may inhibit fungal growth [1,2,87,88]. This condition, combined with prolonged UV exposure during long summer days and low humidity, may contribute to the results obtained, as already reported [2,51].

When analysing the distribution of total fungal counts by region at coastal beaches, a decrease in recorded values is also observed across mainland Portugal from north to south, in both 2024 and 2025. The northern region presented the highest median values, while the Algarve and Alentejo regions recorded the lowest values, which could be related to climatic differences between the north and the south of the country [59]. This pattern is consistent with findings from a previous study conducted on beaches awarded the Blue Flag [51]. Regarding fungal counts by region, inland beaches did not show the same trends due to the limited number of samples. Furthermore, the values in the northern region (2024 median = 2200 CFU/g; 2025 median= 1950 CFU/g) and Alentejo (2025 median = 2200 CFU/g) are strongly influenced by high fungal counts in exceedance beaches (Figure 2 and Figure 3).

However, the correlation calculated between air humidity recorded on the sampling day and fungal levels presented a low value (ρ = 0.14), indicating that this parameter may not be the best indicator for assessing sediment moisture. For future studies, it is therefore proposed to evaluate rainfall events in the days preceding sampling or to directly measure the percentage of water retained in sand grains to better assess the influence of this parameter on fungal survival. Additionally, future studies would benefit from the overall use of more precise climatic data collected in situ at the time of sampling, rather than regional data obtained from online sources.

The reference value used to establish the criterion applied by the Blue Flag programme was based on a study conducted across several European beaches, which reported a median of 76.7 (0.0–3497.5) CFU/g for coastal beaches and 201.7 (0.0–6400.0) CFU/g for inland beaches [2]. In the present study, the values obtained were 10.0 (0.0–8000.0) CFU/g for coastal beaches and 330.0 (48.0–44,000.0) CFU/g for inland beaches in 2024, and 5.0 (0.0–6500.0) CFU/g and 1950.0 (30.0–14,000.0) CFU/g, respectively, in 2025.

These results highlight a substantial variability between beaches, possibly associated with all the climatic and environmental factors already discussed and possible anthropogenic influences that can influence the presence of microorganisms, discussed later in this section. This variability and heterogeneity of beach sands may also explain the differences observed when comparing values reported in other studies [45,88,89,90], as well as results from earlier research conducted in Portugal [51,91]. Consequently, establishing a single reference value remains challenging. This variability is particularly evident for inland beaches, whose values differ markedly both among sites and between 2024 and 2025, and from those reported in previous studies. For example, one study reported median values of 201.7 (0.0–6400.0) CFU/g for inland beaches [2], while another [73] reported values between 1700 and 2800 CFU/g, both lower than those observed in the present study.

In this context, the work carried out by Portugal’s Blue Flag Association, by including sand monitoring within its criteria, represents an important step towards generating data that may help clarify these differences. However, these comparisons and possible causes for the registered patterns should be interpreted with caution given the descriptive and exploratory nature of the analyses. Although the analysed data are not without limitations and do not support definitive conclusions, they provide a useful basis for identifying broader fungal patterns and exploring possible explanations in an essentially exploratory framework.

### 4.2. Variability in Faecal Contamination Indicators

Regarding faecal contamination indicators, the observed trends differ from those described for total fungal counts. These microorganisms have identical threshold values for both inland and coastal beaches, however the *p*-value (<0.001) indicates statistically significant differences in the distribution of enterococci between the two beach typologies. A tendency towards more consistent enterococci levels was observed at inland beaches, although maximum values were slightly higher at coastal beaches (Figure 2 and Figure 3). The contamination detected at inland beaches may share similar origins with fungal contamination, as the previously mentioned causes promote the proliferation of microorganisms in general [69,70,74,75].

It was observed that enterococci contamination was not always accompanied by *E. coli* contamination, although both are frequently recorded together. The results also showed a correlation coefficient of ρ= 0.29 (for 2024), which, although weak, may reflect this co-occurrence. The lower values of *E. coli* may be explained by the lower environmental resistance to the environmental factors discussed above, leading to the faster decay in these environments [33,75,83].

In many cases, the values recorded in this study were 0 or close to 0, resulting in very low or even null median values across most regions and months. Moreover, in 2025, the beaches presenting non-compliances were mostly different from those identified in 2024. These results suggest that these parameters may be mostly influenced by contamination events, especially in coastal beaches, since these bacteria are not part of the native microbiota of these environments and are therefore introduced through external sources [92].

### 4.3. Importance of Exceedance Events

Fourteen exceedance situations were identified according to the Blue Flag criteria, representing 11.5% of the monitored beaches in 2024 and 10.1% in 2025. Even though relatively few beaches were found to be in exceedance situations, it is important to analyse the parameters contributing to non-compliances, particularly those leading to exceedances. Understanding and highlighting some possible causes of the observed contamination levels may allow these sources to be mitigated or prevented, thereby ensuring a safer environment for beach users.

Special attention should be given to cases where non-compliance is recorded for at least one faecal indicator parameter together with elevated total fungal counts, since a major concern associated with faecal indicator organisms is not the indicators themselves, but the potential presence of pathogens they signal. Therefore, the fungal counts recorded may not necessarily represent natural fluctuations in the beach environment and, similarly to the analysed bacteria, may have been introduced through external contamination sources that contribute to the growth of fungi associated with faecal pollution, including clinically relevant species [44,64,93,94,95]. Analysis of the 2025 data also revealed a positive correlation between total fungal counts and enterococci (ρ = 0.23), suggesting that the origin of some recorded contamination events may simultaneously contribute to increases in both indicators during the analysed period.

### 4.4. Possible Sources of Contamination—Wastewater Treatment Plants (WWTPs)

A possible cause of the faecal contamination events recorded in this study is the discharge of sewage and wastewater into these environments. An analysis of the number of public urban WWTPs in Portugal by treatment level indicates that most are equipped with secondary treatment (57%), followed by more advanced treatment processes (41%) [96].

However, these WWTPs may still represent a potential source of contamination for these environments given that secondary treatment does not remove the entire microbial load (approximately 85–95%) [97]. It is also important to note that such infrastructures may also experience operational failures, even with modern equipment. WWTPs may become overloaded or suffer damage following heavy rainfall storm events releasing untreated effluents into the environment, contributing to contamination episodes at beaches [98].

In May 2024, three beaches located in Algarve, were affected by enterococci contamination. Analysis of bathing water profiles indicated that the most probable contamination sources were failures in sanitation infrastructures or malfunctioning stormwater drainage systems, particularly during periods of intense precipitation, such as that recorded in May. For several coastal beaches previously identified as exceeding the established thresholds, bathing water profiles also indicate that sanitation networks and sewage systems are the most probable causes of the contamination events [99]. These factors could possibly explain some of the contamination events recorded for faecal indicator parameters, although in situ assessment would be required to confirm the validity of this interpretation.

In addition, sanitary facilities located directly on beaches must also be considered as potential localised sources of pollution, as these infrastructures are often situated within the beach sand area, failures or inadequate sanitation systems may represent a risk, which may be exacerbated by increased beach attendance and intensive use of these facilities, leading to higher concentrations of faecal pollution in the sand [100,101,102,103].

In August 2025, an outbreak of gastroenteritis was reported in the Centre region following a discharge of untreated sewage caused by a technical failure in the drainage system. The event affected more than 70, who presented typical gastrointestinal infection symptoms such as nausea, vomiting, diarrhoea and headaches [104]. This event combined with the outbreak reported in June 2019 in Azores, in which several beach users developed skin rashes after contact with sand [105], represents clear examples of the vulnerability of bathing areas to technical failures in sanitation and drainage systems, with direct impacts on both beach sand and public health [100,101,102,103].

### 4.5. Human Presence

Human presence at beaches may also contribute to the elevated contamination levels in beach sand, mainly due to the increased use of existing facilities, such as sanitary infrastructures and surrounding bars, as well as the deposition of organic residues from food in the sand, the accumulation of litter [33,73,100,106,107]. The presence of domestic animals and synanthropic birds such as gulls or pigeons, which are often attracted by food waste or by proximity to urban areas, may also act as vectors of pathogenic microorganisms and introduce faecal organisms to the sand by direct deposition of faeces [108]. These organic residues and microorganisms may persist in the sand, even with regular beach cleaning [84,101].

In 2024 and 2025, higher tourist attendance in Portugal was recorded during July and August, corresponding to the typical peak of the summer season [109,110]. However, exceedance events at coastal beaches appear to occur throughout the analysed period. While a direct causal relationship between these factors and specific exceedance events cannot be established, their cumulative, interacting, and potentially persistent effects mean that they should be considered when interpreting exceedance patterns.

### 4.6. Bathing Water

When comparing data published by the Portuguese Environment Agency (APA) regarding bathing water quality during the 2024 bathing season, it can be observed that most exceedances and bathing prohibitions occurred in the water and were not reflected in sand analysis results [111]. This can suggest that these environments are relatively independent and may be influenced by different contamination sources and factors, highlighting the importance of sand monitoring alone [112].

Nevertheless, some bathing prohibitions occurred around the same period as exceedances detected in the sand. Some beaches exhibited non-compliances around the time the corresponding coastal areas were closed for bathing due to the presence of faecal indicators [111]. These data suggest that some contamination events may have resulted from a common source simultaneously. An example of this possibility is the case of a beach in the Centre region in July, where the non-compliance recorded in sand samples may be associated with the opening of the river mouth to the sea. This environmental factor may directly affect both water and sand and alter the composition of microbial communities, particularly fungal communities [73,74,92].

Some coastal beaches identified as exceedance cases, which recorded non-compliances either for fungal counts alone or together with faecal indicators, also presented river or stream discharges in their bathing water profiles. These freshwater inputs are usually identified as the most plausible sources of the contamination observed, according to the bathing water profile [99]. In 2025, a general decline in water quality was also observed, particularly in the northern region. This situation could be related to the higher proportion of samples in the North region presenting non-compliances compared with other regions [96].

The values recorded for microorganisms at a given beach may be influenced by multiple factors, such as those discussed throughout this study, whose combined effects may be reflected in the high variability observed between beaches or even samples of the same beach. Interpreting the contamination patterns observed, as well as discussing the possible factors underlying them, remains challenging. Although several known possible causes for the contamination and values recorded in this study are presented throughout this discussion, it is not possible to state with certainty which factors are driving the observed patterns. A detailed in situ assessment would be necessary to better understand whether the contamination originated from punctual sources or from diffuse pollution.

Even though the large scope of this study constituted a positive aspect that contributed to its statistical strength, it also led to several limitations that should be addressed. These include some degree of inter-laboratory or inter-method variability across sampling sites and laboratories and the external generation of a substantial proportion of the analytical dataset, that made analytical harmonization difficult. In addition, the assessment of climatic influences was based on regional meteorological data obtained from online sources rather than site-specific in situ measurements which may have reduced the accuracy and reliability of the analysed data including the correlation analyses that were exploratory in nature and should not be interpreted as evidence of causality. Additionally, the imbalance between coastal and inland beaches, together with the smaller number of inland samples, also reduced the statistical power of some comparisons between typology and observed patterns.

### 4.7. Emerging Challenges

Based on previous studies on the fungal composition of beach sands, it is possible to identify groups of fungi that constitute the characteristic mycological fauna of beach sand such as *Aspergillus* spp., *Penicillium* spp., *Cladosporium* spp., *Fusarium* spp., *Mucor* spp. and *Rhizopus* spp. Therefore, it is important to emphasise that very high fungal counts are not necessarily associated with a direct source of pollution [45,65,88,112]. It may be concluded that even in the absence of external contamination sources, the natural fluctuation of the resident fungal flora may itself constitute a risk factor since many of these fungi are associated with cutaneous, respiratory, systemic or allergic infections, particularly in immunocompromised individuals [113].

Other fungi may also be frequently found in beach sand, although generally at lower concentrations and more often associated with contamination sources, such as *Candida* spp., and dermatophytes such as *Trichophyton* and *Microsporum*, which are mainly transmitted by direct contact or through contact with contaminated sand [93,94,95,114,115].

This variability for fungi makes precise modelling of sandy environments particularly difficult, especially in the absence of relevant prior quantitative data. At present, the threshold values adopted by the Blue Flag Programme should therefore be used only as a general starting point, and additional studies with a more specific geographical focus and comparable methodologies are recommended. In contrast, the limit set for enterococci is already associated with an increased health risk [26]. In this context, and despite the absence of a specific legal framework for microbiological monitoring of beach sand, the results obtained reinforce the relevance of voluntary initiatives such as those promoted by the Blue Flag Programme in Portugal.

More broadly, traditional faecal indicators alone may be insufficient to capture the diversity of microorganisms relevant to public health [37,44,94,116]. For future studies the integrated consideration of multiple environmental and microbiological parameters is essential for a robust assessment providing a more accurate estimate of the real risk to which beach users are exposed [116,117,118,119]. The use of other potential indicators such as *Candida* species (such as *C. albicans*, *C. tropicalis*, *C. krusei* and *C. glabrata*) has been widely recommended because of their direct association with faecal and anthropogenic contamination and their clinical relevance [21,93,94,95,120]. This should be complemented by in situ assessment of human attendance, bathing water quality, as well as beach cleanliness and support infrastructures such as sanitary facilities, bars and waste collection points, which often constitute critical nodes in contamination [1,42,49,71].

Climate change and the new challenges associated with urbanisation are also imposing a new reality on bathing areas. Global warming promotes the expansion of thermotolerant and potentially pathogenic microorganisms, while also favouring the adaptation of certain fungal species to higher temperatures, potentially enabling them to tolerate conditions closer to human body temperature and thereby increasing their capacity to infect humans [121,122,123,124,125]. At the same time, anthropogenic pressures such as heavy metal contamination and intensive azole use in agriculture may promote the selection of antifungal-resistant strains and alter the fungal community [126,127,128,129,130,131]. Additional emerging challenges include the spread of invasive species such as the alga *Rugulopteryx okamurae*, which has recently, especially in 2025, become frequent on Portuguese beaches and may have increased organic matter accumulation in sand, potentially favouring microbial and fungal growth [132]. This is reshaping fungal ecology, leading to infections that are more frequent, more severe and more difficult to treat, which could make fungal infections one of the major public health challenges of the coming decades [52,121,123].

## 5. Conclusions

Reflecting on the results obtained in this study, it can be concluded that microbiological analysis and continuous monitoring of beach sands are not only feasible, but also essential for understanding the trends recorded on Portuguese beaches. Across the two analysed periods, the sands of the beaches awarded the Blue Flag do not present major sanitary risks, with only 14 of 122 in 2024 and 15 of 149 beaches in 2025 exceeding the limits more than once. Although the analysed beaches did not show many contamination events involving faecal contamination indicators, this study still demonstrates the presence of these bacteria in Portuguese beach sands, with 23 beaches in 2024 and 16 in 2025 exceeding the established limits for enterococci, which is directly associated with an increased public health risk.

Total fungal counts on coastal beaches appear to be influenced by climatic factors, particularly rainfall, while fungal communities at inland beaches showed greater variability, which could be related to smaller sample size or local environmental characteristics such as sediment origin, erosion processes, vegetation and beach morphology. More broadly, the values recorded at a given beach may reflect the combined influence of multiple climatic, environmental, and anthropogenic factors, which are highly relevant when attempting to identify a pattern or trend for observed or expected values, making it particularly difficult to compare different beaches and define a reference value. Although this study does not allow for definitive conclusions regarding specific patterns or their underlying causes, it provides a valuable exploratory basis for identifying broader trends in fungi and faecal indicator bacteria, and for guiding future research.

Furthermore, this study highlights the role of fungi as potential pathogenic agents, an emerging issue that remains insufficiently explored, demonstrating the importance of including fungi in sand monitoring programmes while drawing attention to the need to include other organisms alongside faecal indicators for a broader view of the sanitary risk associated with these recreational environments.

Ultimately, this study reinforces the need to investigate in greater depth the health risk of pathogenic fungi, including *Aspergillus fumigatus*, *Cryptococcus gattii* and *Candida* spp., and it also alerts us to the potential presence of other agents in these environments, such as parasites, that merit further investigation. This, together with integration of multiple levels of information and action, linking scientific research, coastal management, and the participation of entities responsible for public health and environmental quality, provides a more informed perspective on, and understanding of, these recreational areas.

## Figures and Tables

**Table 1:** Descriptive analyses of 2024 and 2025 bathing season data by beach typology (coastal and inland beaches).

		Total	Coastal	Inland	*p* Value *
**2024**	**Total fungal counts (CFU/g)**				<0.001
	Samples	560	508	52	
	Median (min, max)	15.0 (0.0, 44,000.0)	10.0 (0.0, 8000.0)	330.0 (48.0, 44,000.0)	
	Q1, Q3	2.0, 98.5	2.0, 55.8	190.0, 1257.5	
	**Enterococci (MPN/g)**				<0.001
	Samples	557	505	52	
	Median (min, max)	0.0 (0.0, 9600.0)	0.0 (0.0, 9600.0)	2.0 (0.0, 2000.0)	
	Q1, Q3	0.0, 1.0	0.0, 1.0	0.0, 10.0	
	***Escherichia coli*** **(MPN/g)**				0.404
	Samples	556	504	52	
	Median (min, max)	0.0 (0.0, 1187.0)	0.0 (0.0, 1187.0)	0.0 (0.0, 60.0)	
	Q1, Q3	0.0, 0.0	0.0, 0.0	0.0, 0.0	
**2025**	**Total fungal counts (CFU/g)**				<0.001
	Samples	730	681	49	
	Median (min, max)	13.0 (0.0, 14,000.0)	10.0 (0.0, 6500.0)	1100.0 (30.0, 14,000.0)	
	Q1, Q3	2.0, 70.0	2.0, 53.0	230.0, 2400.0	
	**Enterococci (MPN/g)**				<0.001
	Samples	730	681	49	
	Median (min, max)	0.0 (0.0, 2400.0)	0.0 (0.0, 2400.0)	2.0 (0.0, 687.0)	
	Q1, Q3	0.0, 1.0	0.0, 1.0	0.0, 10.0	
	***Escherichia coli*** **(MPN/g)**				0.858
	Samples	730	681	49	
	Median (min, max)	0.0 (0.0, 300.0)	0.0 (0.0, 222.0)	0.0 (0.0, 300.0)	
	Q1, Q3	0.0, 0.0	0.0, 0.0	0.0, 0.0	

* Wilcoxon rank sum test. **Note:** Bold text is used to identify category headings or grouped variables only and does not indicate statistical significance or highlighted values.

**Table 2:** Total precipitation and Mean Temperature over the course of the analysed months.

Year	Month	Total Precipitation (mm)	Mean Temperature (°C)
2024	April	43.5	15.45
	May	33.5	16.59
	June	42.2	19.98
	July	10.1	23.15
	August	0.7	23.85
	September	32.9	19.73
	October	148.7	17.52
2025	May	42.3	17.32
	June	4.9	22.49
	July	3.3	23.65
	August	3.0	24.40
	September	25.8	20.1

## Data Availability

The original contributions presented in this study are included in the article/supplementary material. Further inquiries can be directed to the corresponding author.

## Supplementary Materials

The following supporting information can be downloaded at https://www.mdpi.com/article/10.3390/microorganisms14051043/s1: Table S1. Descriptive analyses of 2024 bathing season data by beach typology (coastal and inland beaches) and region in Portugal; Table S2. Descriptive analyses of 2025 bathing season data by beach typology (coastal and inland beaches) and region in Portugal; Figure S1. 2024 bathing season correlations plots; Figure S2. 2025 bathing season correlations plots; Table S3. Number of compliant and non-compliant samples of 2024 bathing season, according to the proposed limits by ABAAE (Total Fungal Count: Coastal-420 CFU/g, Inland-1130 CFU/g; Enterococci-60 MPN/g; *Escherichia coli*-25 MPN/g); Table S4. Number of compliant and non-compliant samples of 2025 bathing season, according to the proposed limits by ABAAE (Total Fungal Count: Coastal-420 CFU/g, Inland-1130 CFU/g; Enterococci-60 MPN/g; *Escherichia coli*-25 MPN/g); Figure S3. Image of fungal colonies grown on Petri dishes containing malt extract agar supplemented with chloramphenicol; Figure S4. Image of fungal colonies grown on Petri dishes containing malt extract agar supplemented with chloramphenicol.

## Abbreviations

The following abbreviations are used in this manuscript:

CFU/g	Colony Forming Units per gram
MPN/g	Most Probable Number per gram
