# Supplementary material for: Sand Quality on Portuguese Blue Flagged Beaches: Fungal and Faecal Contamination Across Two Bathing Seasons
Source: Microorganisms. 2026 May 5;14(5):1043. doi: 10.3390/microorganisms14051043 (PMC13210291; doi:10.3390/microorganisms14051043)
Supplement: Supplementary file 1 [file microorganisms-14-01043-s001.zip › microorganisms-4239591-supplementary.pdf]

Table S1. Descriptive analyses of 2024 bathing season data by beach typology (coastal and inland beaches) and regions of Portugal

REGION		COASTAL (N=508)	INLAND (N=52)	P VALUE
NORTH	**Total fungal counts (CFU/g)**			< 0.001[1]
	Samples	47	18	
	Median (min-max)	140.0 (0.0, 2300.0)	2200.0 (52.0, 44000.0)	
	Q1,Q3	44.0, 326.0	309.2, 5700.0	
	**Enterococci (MPN/g)**			0.239[1]
	Samples	47	18	
	Median (min-max)	9.0 (0.0, 1700.0)	2.5 (0.0, 201.0)	
	Q1,Q3	0.0, 15.0	0.0, 5.8	
	Escherichia coli **(MPN/g)**			0.124[1]
	Samples	46	18	
	Median (min-max)	0.0 (0.0, 75.0)	0.0 (0.0, 2.0)	
	Q1,Q3	0.0, 1.0	0.0, 0.0	
CENTER	**Total fungal counts (CFU/g)**			< 0.001[1]
	Samples	64	15	
	Median (min-max)	39.0 (0.0, 4500.0)	160.0 (48.0, 360.0)	
	Q1,Q3	6.5, 85.5	99.0, 255.0	
	**Enterococci (MPN/g)**			< 0.001[1]
	Samples	64	15	
	Median (min-max)	0.0 (0.0, 43.0)	6.0 (0.0, 2000.0)	
	Q1,Q3	0.0, 0.0	0.0, 46.5	
	Escherichia coli **(MPN/g)**			0.142[1]
	Samples	64	15	
	Median (min-max)	0.0 (0.0, 14.0)	0.0 (0.0, 60.0)	
	Q1,Q3	0.0, 0.0	0.0, 0.0	
LISBON AND TAGUS VALLEY	**Total fungal counts (CFU/g)**			0.017[1]
	Samples	152	2	
	Median (min-max)	9.0 (0.0, 8000.0)	1610.0 (1210.0, 2010.0)	
	Q1,Q3	2.0, 50.8	1410.0, 1810.0	
	**Enterococci (MPN/g)**			0.877[1]
	Samples	149	2	
	Median (min-max)	0.0 (0.0, 9600.0)	0.5 (0.0, 1.0)	
	Q1,Q3	0.0, 1.0	0.2, 0.8	
	Escherichia coli **(MPN/g)**			0.492[1]

	Samples	149	2	
	Median (min-max)	0.0 (0.0, 410.0)	0.0 (0.0, 0.0)	
	Q1,Q3	0.0, 0.0	0.0, 0.0	
**ALENTEJO**	**Total fungal counts (CFU/g)**			< 0.001[1]
	Samples	77	17	
	Median (min-max)	5.0 (0.0, 1000.0)	330.0 (150.0, 1400.0)	
	Q1,Q3	0.0, 33.0	270.0, 680.0	
	**Enterococci (MPN/g)**			< 0.001[1]
	Samples	77	17	
	Median (min-max)	0.0 (0.0, 4.0)	2.0 (0.0, 200.0)	
	Q1,Q3	0.0, 0.0	0.0, 6.0	
	Escherichia coli **(MPN/g)**			0.598[1]
	Samples	77	17	
	Median (min-max)	0.0 (0.0, 1.0)	0.0 (0.0, 5.0)	
	Q1,Q3	0.0, 0.0	0.0, 0.0	
**ALGARVE**	**Total fungal counts (CFU/g)**			
	Samples	148	0	
	Median (min-max)	5.0 (0.0, 480.0)	NA	
	Q1,Q3	0.0, 20.5	NA	
	**Enterococci (MPN/g)**			
	Samples	148	0	
	Median (min-max)	0.0 (0.0, 230.0)	NA	
	Q1,Q3	0.0, 1.0	NA	
	Escherichia coli **(MPN/g)**			
	Samples	148	0	
	Median (min-max)	0.0 (0.0, 1187.0)	NA	
	Q1,Q3	0.0, 0.0	NA	
**AZORES**	**Total fungal counts (CFU/g)**			
	Samples	15	0	
	Median (min-max)	10.0 (1.6, 58.0)	NA	
	Q1,Q3	3.3, 11.5	NA	
	**Enterococci (MPN/g)**			
	Samples	15	0	
	Median (min-max)	0.9 (0.0, 10.0)	NA	
	Q1,Q3	0.1, 10.0	NA	
	Escherichia coli **(MPN/g)**			

	Samples	15	0
	Median (min-max)	0.0 (0.0, 10.0)	NA
	Q1,Q3	0.0, 10.0	NA
**MADEIRA**	**Total fungal counts (CFU/g)**		
	Samples	5	0
	Median (min-max)	7.0 (0.0, 63.0)	NA
	Q1,Q3	2.0, 47.0	NA
	**Enterococci (MPN/g)**		
	Samples	5	0
	Median (min-max)	0.0 (0.0, 9.0)	NA
	Q1,Q3	0.0, 0.0	NA
	***Escherichia coli* (MPN/g)**		
	Samples	5	0
	Median (min-max)	0.0 (0.0, 0.0)	NA
	Q1,Q3	0.0, 0.0	NA

1. **Wilcoxon rank sum test**

Table S2. Descriptive analyses of 2025 bathing season data by beach typology (coastal and inland beaches) and Portugal's Regions

REGION		COASTAL (N=681)	INLAND (N=49)	P VALUE
**NORTH**	**Total fungal counts (CFU/g)**			< 0.001[1]
	Samples	104	10	
	Median (min-max)	65.0 (5.0, 6500.0)	1950.0 (250.0, 4400.0)	
	Q1,Q3	14.5, 137.2	1525.0, 2400.0	
	**Enterococci (MPN/g)**			0.064[1]
	Samples	104	10	
	Median (min-max)	1.0 (0.0, 2400.0)	5.0 (1.0, 58.0)	
	Q1,Q3	0.0, 14.2	3.2, 7.0	
	***Escherichia coli* (MPN/g)**			0.091[1]
	Samples	104	10	
	Median (min-max)	0.0 (0.0, 80.0)	0.0 (0.0, 0.0)	
	Q1,Q3	0.0, 0.0	0.0, 0.0	
**CENTER**	**Total fungal counts (CFU/g)**			< 0.001[1]
	Samples	61	17	

	Median (min-max)	30.0 (0.0, 480.0)	220.0 (43.0, 14000.0)	
	Q1,Q3	12.0, 68.0	180.0, 230.0	
	**Enterococci (MPN/g)**			0.088[1]
	Samples	61	17	
	Median (min-max)	0.0 (0.0, 20.0)	0.0 (0.0, 687.0)	
	Q1,Q3	0.0, 0.0	0.0, 10.0	
	***Escherichia coli* (MPN/g)**			0.631[1]
	Samples	61	17	
	Median (min-max)	0.0 (0.0, 21.0)	0.0 (0.0, 10.0)	
	Q1,Q3	0.0, 0.0	0.0, 0.0	
**LISBON AND TAGUS VALLEY**	**Total fungal counts (CFU/g)**			0.084[1]
	Samples	162	2	
	Median (min-max)	9.5 (0.0, 719.0)	1092.5 (30.0, 2155.0)	
	Q1,Q3	1.2, 37.0	561.2, 1623.8	
	**Enterococci (MPN/g)**			0.438[1]
	Samples	162	2	
	Median (min-max)	0.0 (0.0, 152.0)	1.0 (0.0, 2.0)	
	Q1,Q3	0.0, 1.0	0.5, 1.5	
	***Escherichia coli* (MPN/g)**			0.641[1]
	Samples	162	2	
	Median (min-max)	0.0 (0.0, 222.0)	0.0 (0.0, 0.0)	
	Q1,Q3	0.0, 0.0	0.0, 0.0	
**ALENTEJO**	**Total fungal counts (CFU/g)**			< 0.001[1]
	Samples	84	20	
	Median (min-max)	8.0 (0.0, 570.0)	2200.0 (230.0, 8000.0)	
	Q1,Q3	0.0, 43.2	940.0, 3800.0	
	**Enterococci (MPN/g)**			< 0.001[1]
	Samples	84	20	
	Median (min-max)	0.0 (0.0, 100.0)	3.5 (0.0, 73.0)	
	Q1,Q3	0.0, 0.0	1.0, 16.5	
	***Escherichia coli* (MPN/g)**			0.313[1]
	Samples	84	20	
	Median (min-max)	0.0 (0.0, 4.0)	0.0 (0.0, 300.0)	
	Q1,Q3	0.0, 0.0	0.0, 0.0	
**ALGARVE**	**Total fungal counts (CFU/g)**			
	Samples	164	0	

	Median (min-max)	3.0 (0.0, 1700.0)	NA
	Q1,Q3	0.0, 18.5	NA
	**Enterococci (MPN/g)**		
	Samples	164	0
	Median (min-max)	0.0 (0.0, 22.0)	NA
	Q1,Q3	0.0, 1.0	NA
	***Escherichia coli* (MPN/g)**		
	Samples	164	0
	Median (min-max)	0.0 (0.0, 4.1)	NA
	Q1,Q3	0.0, 0.0	NA
**AZORES**	**Total fungal counts (CFU/g)**		
	Samples	87	0
	Median (min-max)	7.0 (0.0, 468.0)	NA
	Q1,Q3	2.0, 40.0	NA
	**Enterococci (MPN/g)**		
	Samples	87	0
	Median (min-max)	0.0 (0.0, 58.0)	NA
	Q1,Q3	0.0, 0.0	NA
	***Escherichia coli* (MPN/g)**		
	Samples	87	0
	Median (min-max)	0.0 (0.0, 36.0)	NA
	Q1,Q3	0.0, 0.0	NA
**MADEIRA**	**Total fungal counts (CFU/g)**		
	Samples	19	0
	Median (min-max)	2.0 (0.0, 392.0)	NA
	Q1,Q3	0.0, 4.0	NA
	**Enterococci (MPN/g)**		
	Samples	19	0
	Median (min-max)	0.0 (0.0, 2.0)	NA
	Q1,Q3	0.0, 0.0	NA
	***Escherichia coli* (MPN/g)**		
	Samples	19	0
	Median (min-max)	0.0 (0.0, 2.0)	NA
	Q1,Q3	0.0, 0.0	NA

1. **Wilcoxon rank sum test**

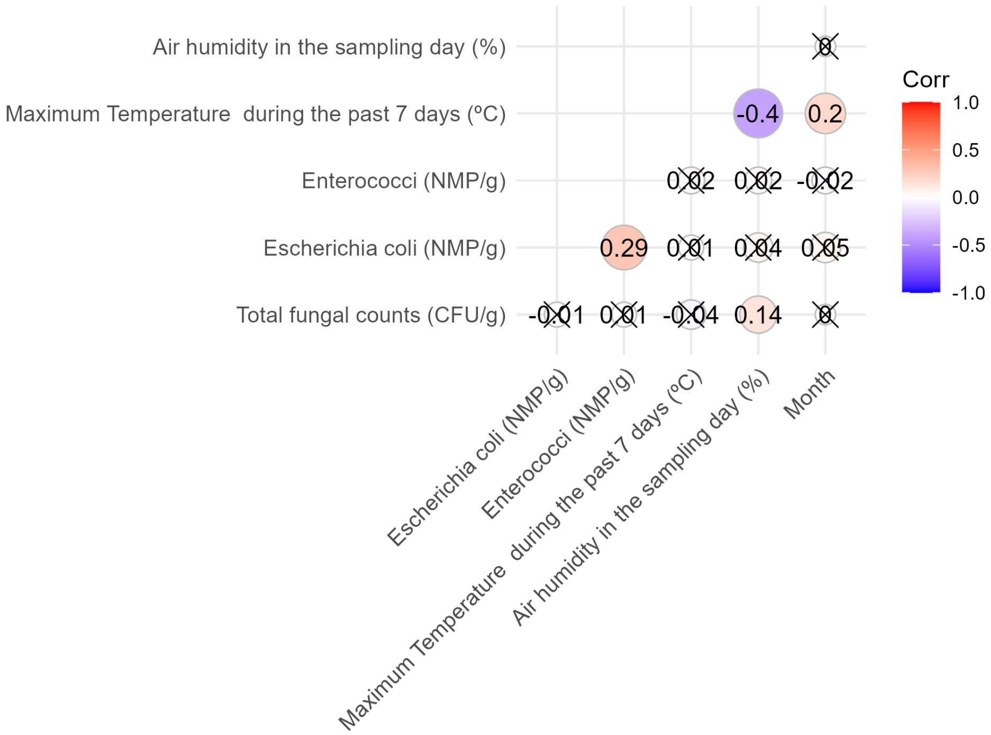

Figure S1. 2024 bathing season correlation plots

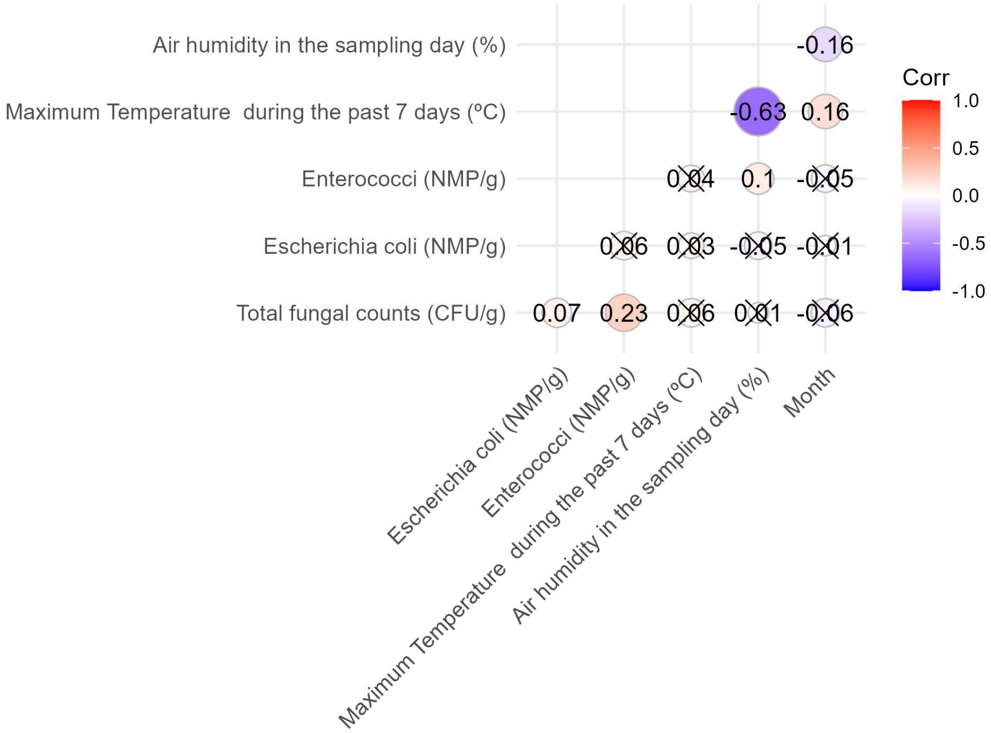

Figure S2. 2025 bathing season correlations plots

Table S3. Number of compliant and non-compliant samples of 2024 bathing season, according to the proposed limits by ABAAE (Total Fungal Count: Coastal-420 CFU/g, Inland-1130 CFU/g; Enterococci-60 MPN/g; *Escherichia coli*-25 MPN/g)

	**Coastal (N=508)**	**Inland (N=52)**
**Total fungal counts (CFU/g)**		
Compliant	481 (94.7%)	37 (71.2%)
No data available	0 (0.0%)	0 (0.0%)
Non-compliant	27 (5.3%)	15 (28.8%)
**Enterococci (MPN/g)**		
Compliant	488 (96.1%)	46 (88.5%)
No data available	3 (0.6%)	0 (0.0%)
Non-compliant	17 (3.3%)	6 (11.5%)
***Escherichia coli* (MPN/g)**		
Compliant	496 (97.6%)	51 (98.1%)
No data available	4 (0.8%)	0 (0.0%)
Non-compliant	8 (1.6%)	1 (1.9%)

Table S4. Number of compliant and non-compliant samples of 2025 bathing season, according to the proposed limits by ABAAE (Total Fungal Count: Coastal-420 CFU/g, Inland-1130 CFU/g; Enterococci-60 MPN/g; *Escherichia coli*- 25 MPN/g)

	**Coastal (N=681)**	**Inland (N=49)**
**Total fungal counts (CFU/g)**		
Compliant	658 (96.6%)	25 (51.0%)
Non-compliant	23 (3.4%)	24 (49.0%)
**Enterococci (MPN/g)**		

Compliant	669 (98.2%)	45 (91.8%)
Non-compliant	12 (1.8%)	4 (8.2%)
***Escherichia coli* (MPN/g)**		
Compliant	675 (99.1%)	47 (95.9%)
Non-compliant	6 (0.9%)	2 (4.1%)

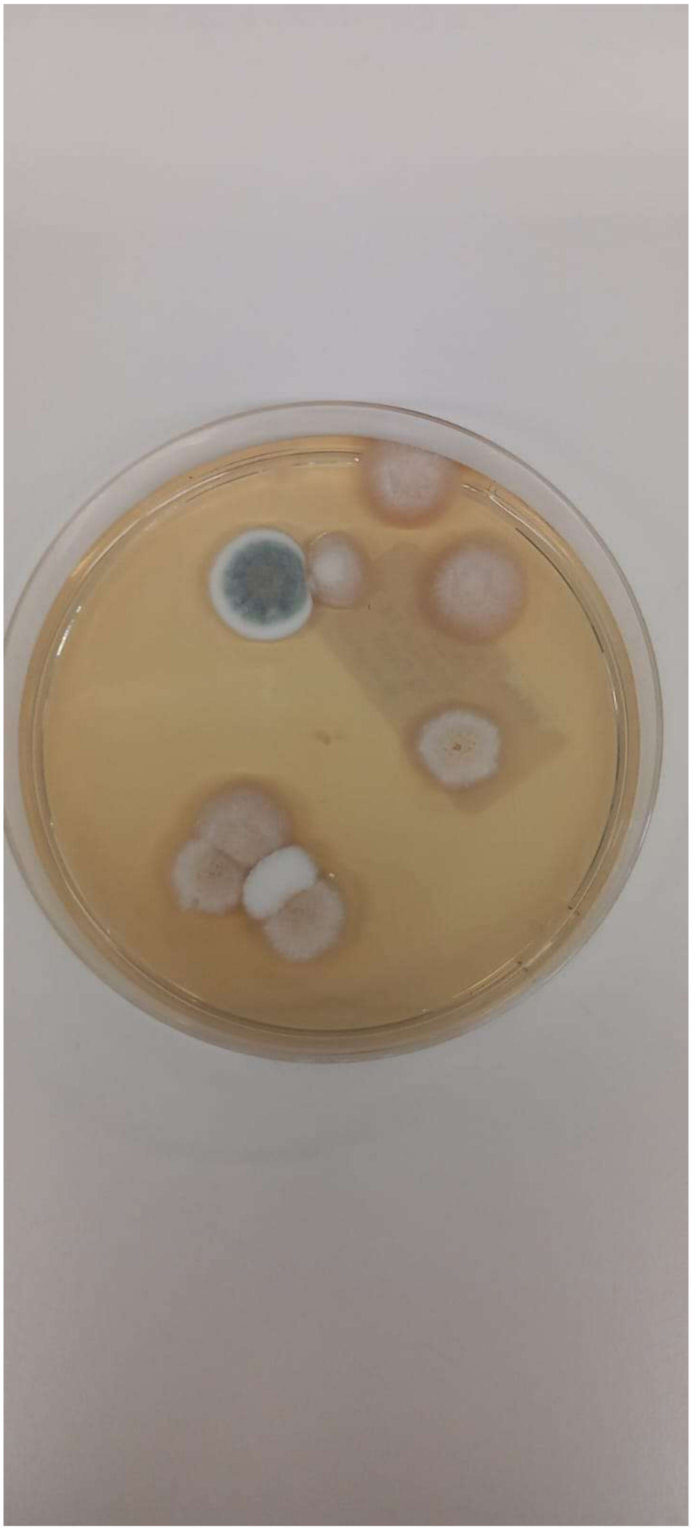

Figure S3. Image of fungal colonies grown on Petri dishes containing malt extract agar supplemented with chloramphenicol

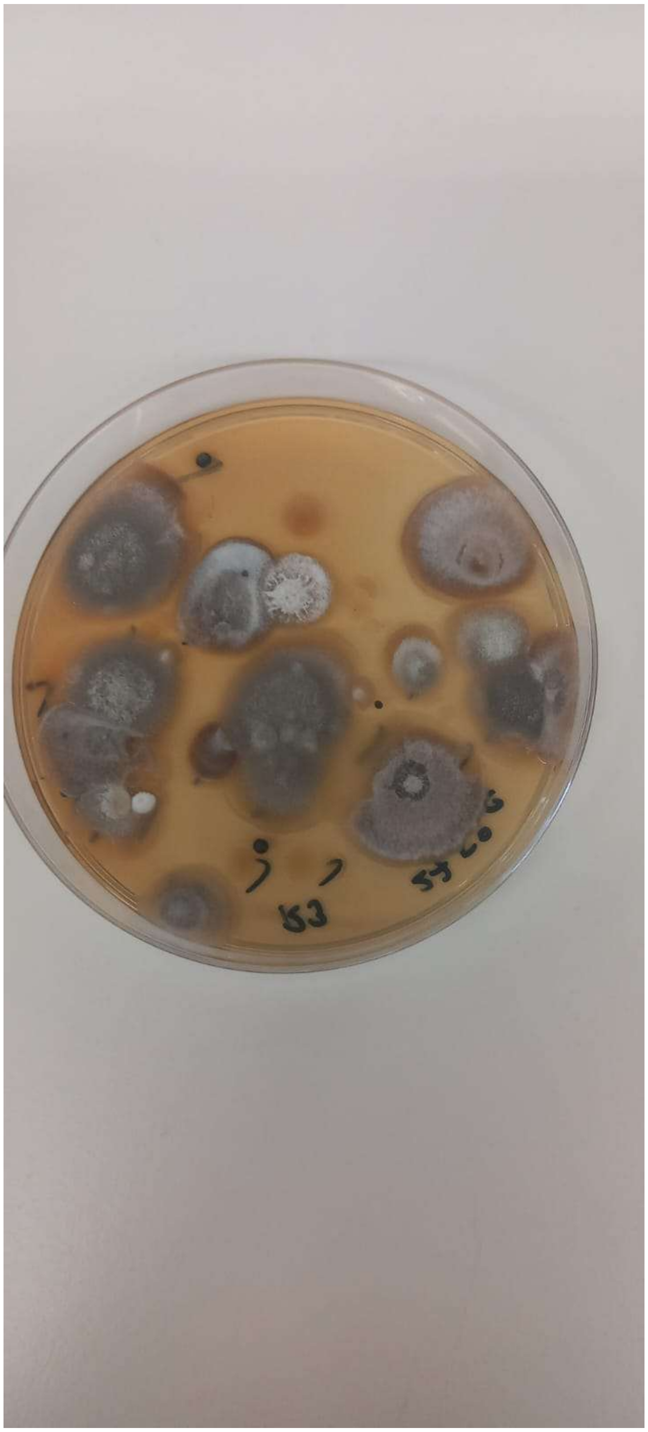

Figure S4. Image of fungal colonies grown on Petri dishes containing malt extract agar supplemented with chloramphenicol
